# Hairy cell leukemia: A retrospective study on 11 patients in the Western of Iran

**Published:** 2015-07-01

**Authors:** Mehrdad Payandeh, Masoud Sadeghi, Edris Sadeghi

**Affiliations:** 1 Department of Hematology and Medical Oncology, Kermanshah University of Medical Sciences, Kermanshah, Iran; 2 Students Research Committee, Kermanshah University of Medical Sciences, Kermanshah, Iran; 3 Medical Biology Research Center, Kermanshah University of Medical Sciences, Kermanshah, Iran

**Keywords:** Hairy Cell Leukemia, Splenomegaly, Cladribine

## Abstract

**Background:** Hairy cell leukemia (HCL) is a chronic B-cell lymphoid leukemia characterized by pancytopenia, splenomegaly, myelofibrosis and the presence in peripheral blood, bone marrow and spleen of atypical lymphoid cells with a hairy aspect. The study aims to evaluate a group of patients with hairy cell leukemia, hospitalized in the Clinic of Hematology and Oncology, Kermanshah, Iran, on a period of 15 years and affect of between cladribine therapy and IFN therapy on the patients with HCL.

**Methods: **This is a retrospective analysis of 11 patients in the Clinic of Hematology and Oncology, Kermanshah, Iran, between 2004 and 2013. Clinical features at diagnosis, differential count (platelet, Hb and WBC) types of therapy, survival rate and BRAF mutation have been monitored. As a result, cladribine therapy is the best treatment option for patients.

**Results: **The mean age of patients was 50 years with 100% of men. Approximately 45% of them had splenomegaly at diagnosis. 100% of patients had pancytopenia at diagnosis.9% of patients had mutation of BRAF V600E. Before of treatment, there were fatigue, weight loss, vomiting, fever, night sweat and itching in all of the patients.

**Conclusion:** There is presence of hairy cells in peripheral blood and bone marrow and was associated with pancytopenia, splenomegaly, myelofibrosis in HCL patients. Also, cladribine therapy is best option for treatment of patients and it is better than IFN.

## Introduction

 Hairy cell leukemia (HCL) is a chronic B-cell lymphoid leukemia characterized by pancytopenia, splenomegaly, myelofibrosis and the presence in peripheral blood, bone marrow and spleen of atypical lymphoid cells with a hairy aspect.^1^ HCL is more frequent in men than in women (4:1 ratio), appearing around 50 years old.^1^ There are approximately 1,000 and 1,600 new cases of HCL per year in the US and Europe, respectively. The prevalence of the disease is unknown, although HCL accounts for 2% of all leukemias, the incidence of HCL increases annually.^2^

The presence of the BRAF V600E mutation has recently been described in hairy cell leukemia (HCL) but not in other common lymphomas^3^ (BRAF V600E mutation has been reported in 50% of melanomas, and is present in more than 85% of HCL cases).^4^

Cladribine is a well-known purine nucleoside analog with activities against lymphoproliferative disorders such as HCL,^5^ and immunomodulatory activities of interferon (IFN) can improve or even normalize peripheral blood counts in patients with HCL.^6^

Currently, flow cytometric analysis (FC) and frozen section immunohistochemistry (IHC) represent the only available methods for diagnosis of hairy cell leukemia (HCL).^7^

Despite the fact that more effective chemotherapeutics are available in the treatment of HCL today, splenectomy still remains a valid treatment option in certain cases. HCL with massive splenomegaly (e.g. >10 cm below costal margin) but low bone marrow involvement can be an indication for splenectomy as first-line treatment. ^6^

Flow cytometric analysis shows the expression of CD103+,^8^ CD11c+, CD19+, CD20+, CD22+^9^ and CD5 in HCL, while CD10- and CD23- are expressed in some cases.^10^

The clinical presentations included splenomegaly, hepatomegaly, fatigue, pallor, weight loss, infections, rarely lymphadenopathy, In 10% of cases, the disease is asymptomatic^1^^,^^11^ but in the rest of cases abdominal pain and the laboratory features such as anemia, monocytopenia and neutropenia were commonly more.^12^

The study aims to evaluate the efficacy of treatment with cladribine and IFN in a group of 11 patients with hairy cell leukemia hospitalized at the Clinic of Hematology and Oncology, Kermanshah, Iran, over a period of 15 years.

## SUBJECTS AND METHODS

 This is a retrospective analysis of 11 patients at the Clinic of Hematology and Oncology, Kermanshah, Iran, between 2004 and 2013. Clinical features including splenomegaly (n=6), fatigue, weight loss, vomiting, fever, pallor, lymphadenopathy were identified in all patients at initial diagnosis. We determined differential count, types of therapy, survival rate and BRAF mutation in all patients. Complete response (CR) to multiple different drug types (consisted of cladribine and/or interferon) was defined as the disappearance of all clinical features, normal spleen size, WBC, hemoglobin and platelet. Partial response (PR) to drugs required the existence of at least clinical features and abnormality in WBC, hemoglobin and platelet. 

**Statistical Analysis**

Survival was determined from date of first administration of cladribine and/or interferon to death. Kaplan-Meier survival  curves (Figure 2) were made with Prism 5 (GraphPad Software). Data (WBC, hemoglobin and platelet) were analyzed using IBM SPSS statistics 19.

## Results

**Figure 1:**
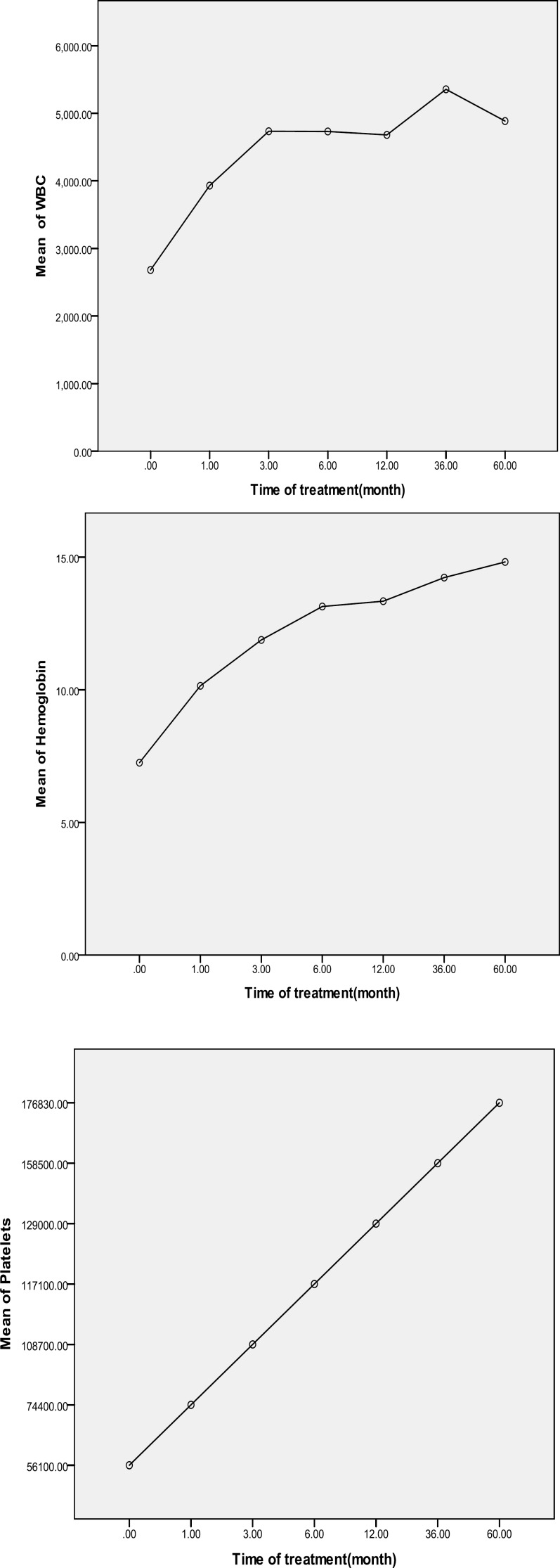
Mean levels of WBC, hemoglobin and platelet at the time of treatment with cladribine and interferon

**Figure 2:**
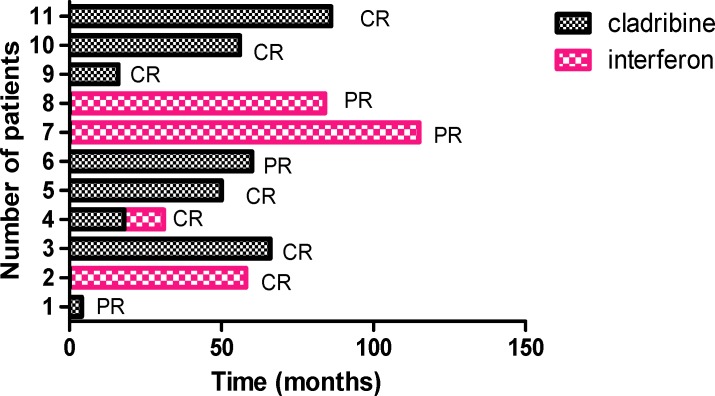
Time after the first course of treatment with drug (cladribine and/or interferon) in 11 patients with hairy cell leukemia, CR: complete response, PR: partial response

 Participants were recruited from males with the mean age of 50 years old. Approximately, 45% of who had splenomegaly at the time of diagnosis (Figure 2), while all patients had pancytopenia at diagnosis. 9% of patients had BRAF V600E mutation (Figure 2). Before treatment, median WBC, hemoglobin and platelets were 2681/µL, 7.25 g.dL^-1^ and 56100 /µL, respectively (Figure 1).

Before treatment, fatigue, weight loss, vomiting, fever, night sweat and itching were reported in all patients. Figure 2 shows the effect of drug treatment in patients after 115 months**.** Three patients (No.2, 7, 8) received interferon alpha, 2 of whom had a PR and 1 had a CR. seven patients (No.1, 3, 5, 6, 9, 10 , 11) received cladribine, 6 of whom had a CR and 1 achieved PR. one patient (No.4) who had received cladribine and interferon showed CR. 

Of the 11 patients, one (No.7) died after 115 months of treatment with interferon. The mean survival time after first administration of cladribine and/or interferon was 56.9 months. The survival rate among patients was 90%.

## Discussion

 Diagnosis of HCL was based on the presence of hairy cells in peripheral blood and bone marrow and was associated with pancytopenia, splenomegaly, myelofibrosis.^1^ Bone marrow, spleen and liver are characteristically infiltrated by leukemic B cells showing abundant cytoplasm with “hairy” projections and unique immunophenotypic features.^13^

In this study, all patients were associated with pancytopenia and splenomegaly, and bone marrow and spleen were infiltrated hairy cells.

In this study, all patients were associated with pancytopenia and splenomegaly, and bone marrow and spleen were infiltrated hairy cells. While little is known about the biological role of 4/5 affected genes, BRAF represents the most frequently mutated gene encoding a protein kinase in human cancers.^14^

Interestingly, the same amino acid change (V600E) identified in our patient represents a mutational hotspot in melanomas and papillary thyroid cancers, where its oncogenic activity has been extensively documented.^15^^,^^16^ Moreover, dysregulation of the mitogen-activated protein kinase (MAPK) pathways, of which BRAF is a component, was previously implicated in HCL pathogenesis.^17^ In conclusion, BRAF V600E mutation seems to have an important role in the pathogenesis of HCL if it is not even the disease-defining genetic event.^6^ 9% of patients had BRAF mutation (n=1). The presence of BRAF mutation in most individuals with HCL and the response of these patients have suggested that BRAF mutations are an oncogenic driver in this disease,^17^ but in this study BRAF V600E mutation appeared not to have very important role in HCL pathogenesis. Hence, in order to achieve a reasonable outcome, it is recommended to recruit more participants from other parts of Iran. 

 In a study on 28 cases of HCL, the median age of patients was 47 years with male predominance (M: F=6:1),^10^ and in another study on 39 HCL patients, the median age of patients was 52 years with a high frequency in men.^1^ This study included only male participants with a median age 50 years**. **According to these studies,

According to 4 earlier studies, men have greater risk of HCL and patients with HCL are usually middle-aged men. 

Cladribine has been identified as a lymphocyte-specific agent^18^ and it induces protracted remissions in patients with hairy cell leukemia. The results indicate that HCL is potentially curable after cladribine treatment.^19^ HCL can be well-controlled with Cladribine: overall complete response rates ranged from 76 to 98%, median disease-free survival of 16 years, normal lifespan and rare HCL-related deaths.^10^ The response of hairy cell leukemia (HCL) to IFN-α is one of the most dramatic and specific effects in the whole of clinical oncology, but the mechanism of action of IFN-α in HCL remains unclear.^20^ Although IFN-α is highly active in HCL, IFN-α is not curative in this disease**.**^21^ Poor response to alpha-IFN-α treatment could likely be related to a peculiar immunological phenotype of the hairy cell.^22^

In this study, 6 of 7 patients treated with cladribine had CR, but among patients (n=3) treated with IFN–α, 1 achieved CR and 1 died after 115 months. The results of a study by Damasio et al on 169 patients showed that the majority of patients (n=169) treated with IFN had PR.^23^ Therefore, IFN therapy cannot be deemed effective in all HCL patients, but cladribine is effective for treatment of patients, and induces high CR rates like the study conducted by Davies et al.^15^

## CONCLUSION

 Diagnosis of HCL is sometimes difficult, but WBC<3000, hemoglobin<10, platelet<100000 and also fatigue, weight loss, vomiting, fever, night sweat and itching in patient are signs of HCL. The presence of hairy cells in peripheral blood and bone marrow was associated with pancytopenia, splenomegaly and myelofibrosis in HCL patients. Moreover, our results have shown that cladribine therapy is the best option for treatment of HCL.
